# Intracranial metastasis from primary transitional cell carcinoma of female urethra: case report & review of the literature

**DOI:** 10.1186/1471-2407-11-23

**Published:** 2011-01-19

**Authors:** Kyung-Sub Moon, Shin Jung, Kyung-Hwa Lee, Eu Chang Hwang, In-Young Kim

**Affiliations:** 1 Department of Neurosurgery, Chonnam National University Research Institute of Medical Sciences, Chonnam National University Hwasun Hospital & Medical School, 160 Ilsim-ri, Hwasun-eup, Hwasun-gun, Jeollanam-do, 519-809, South Korea; 2 Department of Pathology, Chonnam National University Research Institute of Medical Sciences, Chonnam National University Hwasun Hospital & Medical School, 160 Ilsim-ri, Hwasun-eup, Hwasun-gun, Jeollanam-do, 519-809, South Korea; 3 Department of Urology, Chonnam National University Research Institute of Medical Sciences, Chonnam National University Hwasun Hospital & Medical School, 160 Ilsim-ri, Hwasun-eup, Hwasun-gun, Jeollanam-do, 519-809, South Korea

## Abstract

**Background:**

Transitional cell carcinoma (TCC) of the female urethra is a rare urological malignancy, and intracranial metastasis of this cancer has not yet been reported in the literature. This review is intended to present a case of multiple intracranial metastasis in a female patient with a remote history of primary urethral TCC.

**Case Presentation:**

A 49-year-old woman, presented with a prolapsed mass in urethral orifice that was diagnosed as primary urethral TCC with distant lung and multiple bone metastases. The patient subsequently underwent chemotherapy under various regimens. A year later, the patient developed headache and vomiting which as was found to be due to multiple intracranial metastasis. The patient underwent surgical resection of the largest lesion located on the cerebellum, and consecutively gamma knife radiosurgery was performed for other small-sized lesions. Pathological examination of the resected mass revealed a metastatic carcinoma from a known urethral TCC. Serial work-up of systemic metastasis revealed concomitant aggravation of lung, spleen, and liver metastasis. The patient died of lung complication 2 months after the diagnosis of brain metastasis.

**Conclusion:**

To the best of our knowledge, this is the first reported case of cerebral metastasis from primary urethral TCC, with pathological confirmation. As shown in intracranial metastasis of other urinary tract carcinoma, this case occurred in the setting of uncontrolled systemic disease and led to dismal prognosis in spite of aggressive interventional modalities.

## Background

Primary urethral carcinoma is a rare entity, accounting for only 0.02% of all female cancers [1]. The overall annual incidence for women is 1.5 per million and it increases progressively with age [2]. Although there have been some debates in the exact proportion of pathological subtypes of female urethral carcinoma, transitional cell carcinoma (TCC) is one of the most frequently observed subtypes [2,3].

Intracranial metastasis from TCC of urinary system is reported as rare events [4]. Majority of the reported cases of intracranial metastasis from TCC originated from bladder and upper urinary tract [5-12]. To the best of our knowledge, intracranial metastasis from primary urethral TCC has not yet been reported in the literature. In this paper, we present an extremely rare case that underwent surgical resection and gamma knife radiosurgery for multiple intracranial metastases originating from primary urethral TCC.

## Case Presentation

**Figure 1:**
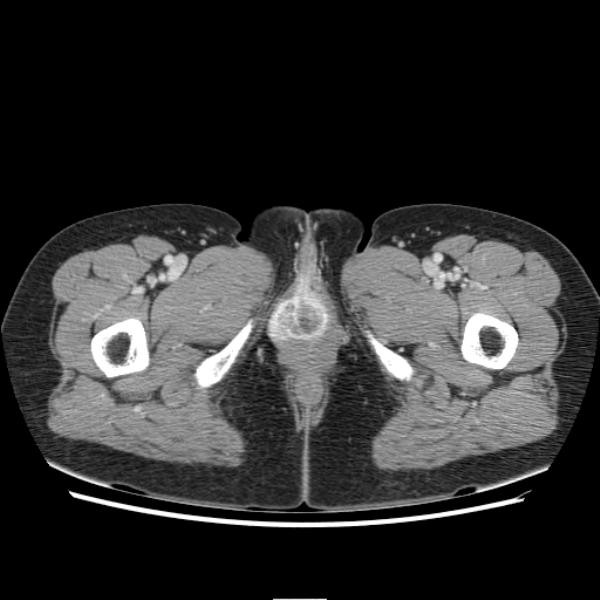
**Enhanced pelvic CT scan demonstrates a heterogeneously enhanced mass on female urethra**.

**Figure 2:**
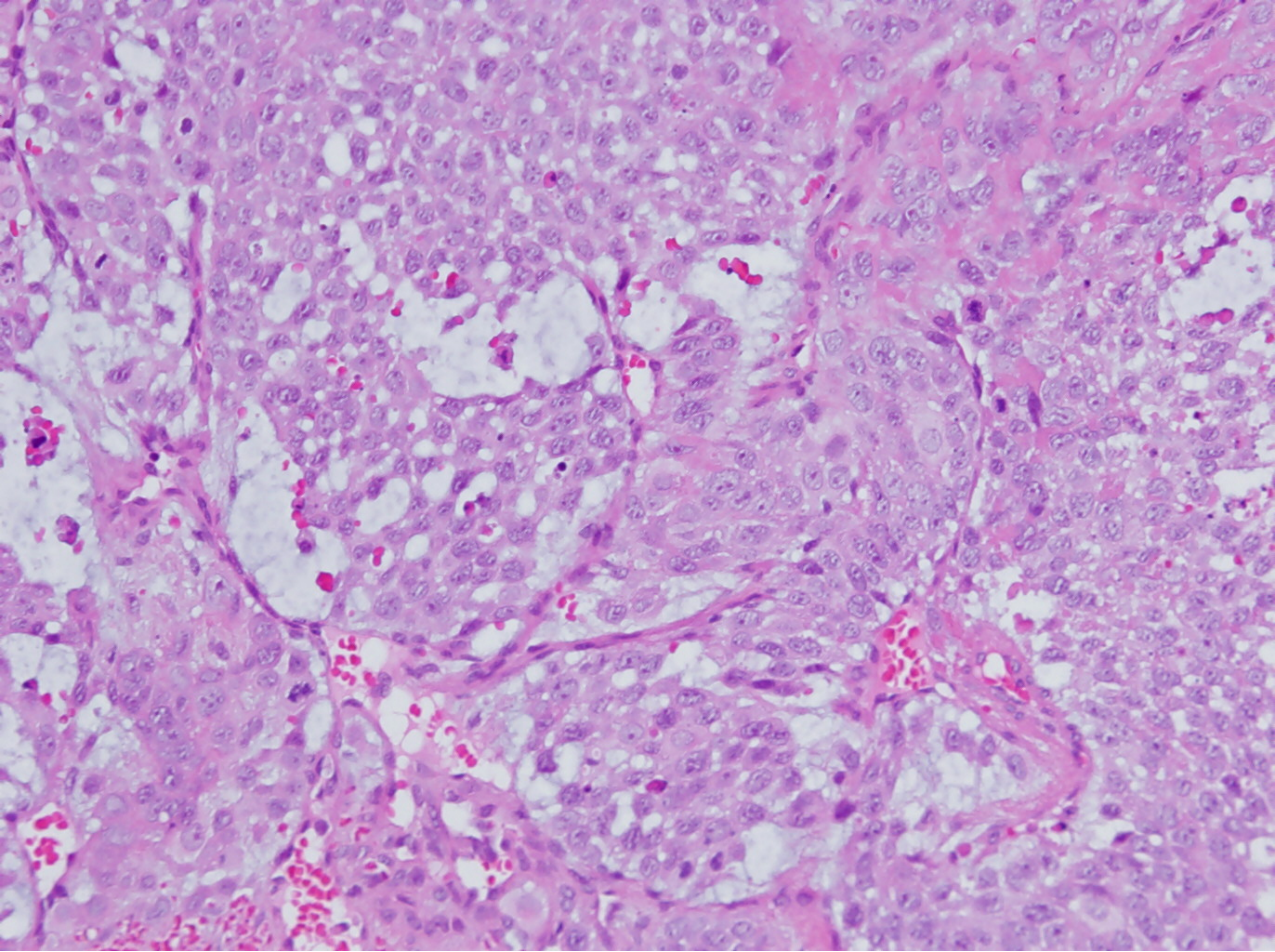
**Microphotograph of the urethral mass revealed typical features of TCC including ramifying papillae, high nuclear/cytoplasmic ratio, and brisk mitotic activity (hematoxylin-eosin, × 200)**.

A 50-year-old woman was admitted with headache and vomiting for 2 days. One year prior to the present admission, she had visited the department of urology for the treatment of a prolapsed mass in urethral orifice. Cystoscopy revealed a proximal urethral mass with an unremarkable bladder. Based on the pelvic computed tomography (CT) (Figure 1), cystoscopy and biopsy findings (Figure 2), she was diagnosed as a primary urethral TCC. Systemic evaluation including positron emission tomography - CT, bone scan, and magnetic resonance (MR) images revealed distant metastases in both the lungs and in multiple bones. The patient had been treated with five courses of M-VAC chemotherapy protocol (45㎎ methotraxate, 4.5㎎ vinblastin, 45㎎ adriamycin, 110㎎ cisplatin). On follow-up, persistent progression of lung and liver metastasis was observed, thus the chemo-regimens had been changed into the second courses of GC protocol (1600㎎ gemcitabine and 100㎎ cisplatin), followed by the third courses of PC protocol (120㎎ palcitaxel and 100㎎ cisplatin).

**Figure 3:**
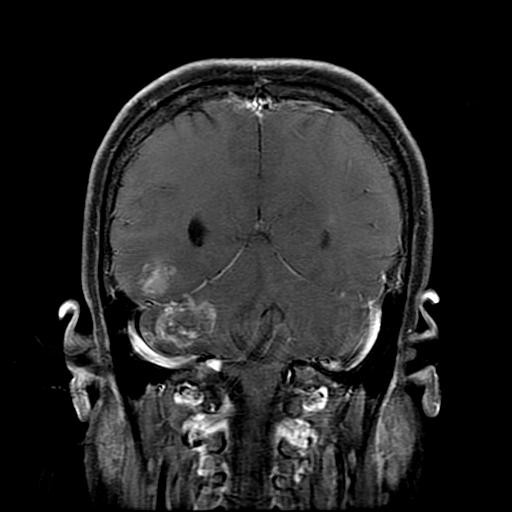
**Brain MR imaging showed multiple heterogeneously enhanced lesions**. The largest lesion, seen in the right cerebellar hemisphere, was later surgically resected.

**Figure 4:**
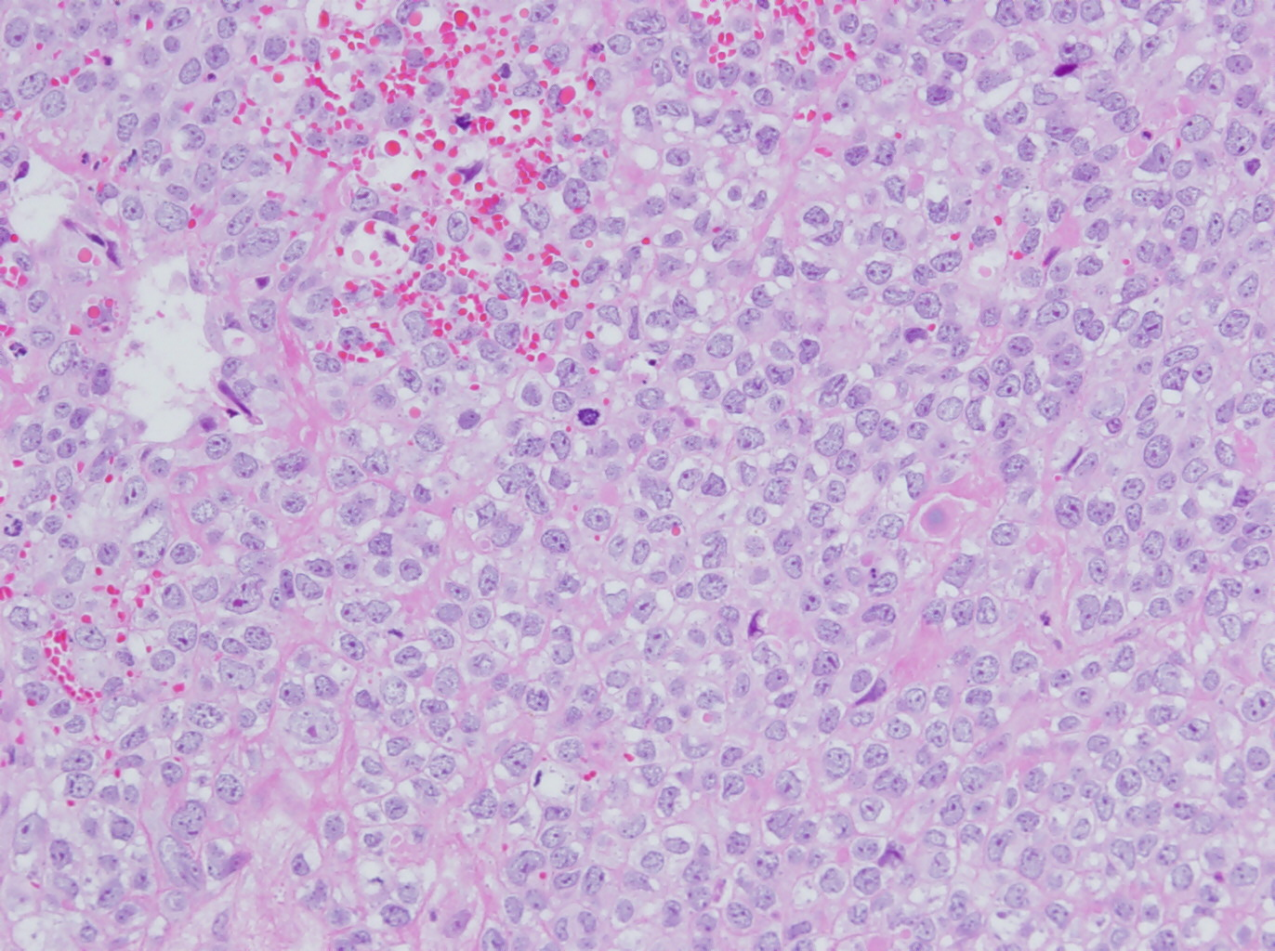
**Microphotograph of the brain mass revealed metastatic carcinoma with increased pleomorphism and mitotic activity which was nearly identical to the urethral mass (hematoxylin-eosin, × 200)**.

On physical examination at the admission, no neurological signs were observed. Brain MR imaging study demonstrated a 3㎝-sized enhanced lesion on the right cerebellar hemisphere with ventricular effacement. Several smaller enhanced lesions were also noted on the cerebral and cerebellar hemisphere. Majority of the lesions showed an internal hyperintensity on the T_1_-weighted MR due to hemorrhage and peritumoral edema as well (Figure 3). After corticosteroid treatment, surgery was performed using a suboccipital approach for the largest lesion on the right cerebellar hemisphere. The mass was removed *in toto *and the postoperative course was uneventful. The pathological findings were consistent with the diagnosis of TCC (Figure 4).

One week after surgery, the patient underwent gamma knife radiosurgery for the remaining multiple metastatic lesions. Systemic re-evaluation revealed concomitant aggravation of lung, spleen, and liver metastases. Although adjuvant chemotherapy was recommended for systemic cancer control, patient's family opted for a conservative medical support. The patient died of aggravation of lung metastasis two months after the diagnosis of brain metastasis.

## Discussion

As per the description of primary urethral carcinoma by Boiven and Deuges published in 1833, these tumors constitute a very small proportion, equivalent to lesser than 1% of all female urothelial cancer [13]. It has been regarded that urethral carcinoma is the only urological cancer that is more common in female than in male [14]. However, in a large population-based study, the incidence of primary urethral carcinoma is relatively higher in men and black population in the United State [2]. The incidence of predominant pathological type in primary urethral carcinoma still remains debatable primarily due to rarity of this entity. In contrary to the previous reports describing that squamous cell carcinoma to be the leading type [14], it has been observed that TCC is encountered more frequently than squamous cell carcinoma and adenocarcinoma [2].

The common mode of metastasis is hematogenous and the frequently observed sites of this metastasis in renal TCC are the lung, liver, lymph node, bone, adrenal glands, spleen, and intestine [15]. Although there is minimal information on the distant metastasis of primary urethral TCC, this cancer would follow the similar characteristics of renal TCC, as shown in a small-patient series [3]. Cerebral metastasis has been reported to be occurring in less than 1% of patients with TCC [7]. Currently, the incidence of cerebral metastasis has increased up to 3% after clinical application of chemotherapy for TCC in the urinary tract system. This could be attributed to the long-term systemic remission that is achieved by chemotherapeutic agents without penetration through the blood-brain barrier [6]. Nevertheless, besides bladder origin, only few reported cases demonstrated cerebral metastasis from TCC in other urinary tract system [5,10,11]. To the best of our knowledge, this is the first reported case of cerebral metastasis from primary urethral TCC, with pathological confirmation.

Based on the review of the literature [5-12], cerebral metastasis presented as one of the features in systemic progression of urothelial TCC but it could occur in the patient with systemic control, and even as an initial manifestation [7,8]. The interval from primary diagnosis to the cerebral metastasis was 0 to 69 months, and it generally developed within 3 years. Although the previous reports have been showing that majority of the reported cases demonstrated multiplicity, solitary cerebral metastasis does not seem to be infrequent [6,8,11,12].

The treatment for cerebral metastasis of TCC may follow the general guidelines as for the metastatic brain tumors. For a single large-sized lesion (> 3cm), especially with a mass effect (> 1cm midline shift), surgical resection should be considered with/without adjuvant whole brain radiotherapy (WBRT). WBRT has been generally accepted as the standard treatment for multiple cerebral metastases. However, considering cognitive deficits after WBRT and the better results of radiosurgery, radiosurgery would play a more important role for the treatment of metastatic brain tumors. Although the role of surgical resection for multiple brain metastases has not been established, surgery has been performed in cases with large lesions or significant mass effects and in cases where two or more lesions are accessible through a single craniotomy approach [16]. Cerebellar metastasis is often more life-threatening than brain metastasis in other locations since it can cause hydrocephalus, irreversible brainstem compression, and tonsilar or upward herniation. Clinical picture could worsen dramatically within a few days and deteriorate acutely on radiation therapy [17]. Although survival has been reported to be worse in the cerebellar metastasis than that in cerebral metastasis [17,18], surgical resection can produce a significant survival gain with clinical improvement [19-21]. Yoshida & Takahashi [21] reported that the median survival of patients who underwent surgical resection and postoperative radiation therapy was 30.5 months, and that the median survival was 20.5 months even for those who received surgical resection only. Despite controversies, such an aggressive local control for cerebellar metastasis may provide a better outcome in those selected patients with single lesion, stable systemic disease, non-lung cancer origin, or high performance status [19-21]. In patients with multiple lesions, poor performance status (KPS < 70) or uncontrolled systemic disease, surgical indications are questionable. Postoperative complication rate has been reported to be relatively high with surgical resection in cases with cerebellar metastasis [20]. This can be explained by the fact that soft and fragile tissue nature of the cerebellum causes difficulty in bleeding control of tumor bed and leads to small-size hematoma or contusion. Such lesions in the supratentorial location are generally negligible from clinical aspects. In a posterior fossa surgery, however, even small increase of volume in space occupying lesion can deteriorate clinical status of patients and result in reoperation.

The current case had showed unfavorable factors in determining surgical resection, such as multiplicity in brain metastasis and uncontrolled systemic disease. Considering the questionable survival benefit and high incidence of postoperative complications, surgical resection for a large cerebellar lesion could seem to be excessive as a treatment option. The current patient was young and regularly received systemic treatments, and, most importantly, demonstrated high performance status without definitive neurological signs. Of aforementioned prognostic factors, high performance status was the most significant parameter in choosing surgical resection for treatment option [20,21]. We authors believed that an aggressive treatment for brain and systemic lesions should not be limited by the occurrence of the cerebellar metastasis especially in patients with good performance. One week after radiosurgery following operation, the patient was discharged home without any neurological deficit. However, the patient did not have the opportunity to get further systemic treatment and died of lung metastasis that had progressed rapidly. Although it is difficult to presume survival benefit of additional systemic chemotherapy in this case, well-controlled brain lesions would allow trying further systemic cancer control.

In selected cases with single brain lesion and well controlled systemic cancer, aggressive treatment including complete surgical resection can prolong the survival and improve the life quality. Nonetheless, once the cerebral metastasis occurred in TCC of urinary tract system, the prognosis is poor, especially in the case with multiple cerebral lesions and/or uncontrolled systemic disease [6,7].

## Conclusion

In conclusion, urethral TCC in female, a very rare cancer, can metastasize to the brain in uncontrolled systemic state and result in dismal prognosis despite of aggressive treatment, as observed in other urinary tract TCC.

## Consent

Written informed consent was obtained from the husband of the patient for publication of this case report and any accompanying images. A copy of the written consent is available for review by the Editor-in-Chief of this journal.

## Abbreviations

CT: computed tomography; TCC: transitional cell carcinoma; MR: magnetic resonance

## Competing interests

The authors declare that they have no competing interests.

## Authors' contributions

KSM & ECH analyzed the data and drafted manuscript. SJ participated in study design and coordination. KHL participated in reviewing literatures and revising the manuscript critically for pathologically content. Kim IY performed the surgical procedure and helped to draft the manuscript. All authors read and approved the final manuscript.

## Pre-publication history

The pre-publication history for this paper can be accessed here:

http://www.biomedcentral.com/1471-2407/11/23/prepub
